# Parent based language intervention for 2-year-old children with specific expressive language delay: a randomised controlled trial

**DOI:** 10.1136/adc.2008.141572

**Published:** 2008-08-14

**Authors:** A Buschmann, B Jooss, A Rupp, F Feldhusen, J Pietz, H Philippi

**Affiliations:** 1 Department of Paediatric Neurology, Children’s Hospital, University of Heidelberg, Heidelberg, Germany; 2 Section of Biomagnetism, Department of Neurology, University of Heidelberg, Heidelberg, Germany; 3 Department of Paedaudiology, University of Heidelberg, Heidelberg, Germany

## Abstract

**Objective:**

The aim of this randomised controlled trial was to evaluate the effectiveness of a short, highly structured parent based language intervention group programme for 2-year-old children with specific expressive language delay (SELD, without deficits in receptive language).

**Methods:**

61 children with SELD (mean age 24.7 months, SD 0.9) were selected between October 2003 and February 2006 during routine developmental check-ups in general paediatric practices, using a German parent-report screening questionnaire (adapted from the MacArthur Communicative Development Inventories). Standardised instruments were used to assess the language and non-verbal cognitive abilities of these children and of 36 other children with normal language development (reference group; mean age 24.6 months, SD 0.8). 58 children with SELD were sequentially randomly assigned to an intervention group (n = 29) or a 12-month waiting group (n = 29). In the intervention group, mothers participated in the 3-month Heidelberg Parent-based Language Intervention (HPLI). All children were reassessed 6 and 12 months after pretest. Assessors were blind to allocation and previous results.

**Results:**

47 children were included in the analysis. At the age of 3 years, 75% of the children in the intervention group showed normal expressive language abilities in contrast to 44% in the waiting group. Only 8% of the children in the intervention group versus 26% in the waiting group met the criteria for specific language impairment (t score ⩽35).

**Conclusions:**

By applying the short, highly structured HPLI in children with SELD, the rate of treatment for language impairment at the age of 3 years can be significantly reduced.

With a prevalence of about 15% language delay is one of the most frequent developmental problems in 2-year-old children.^1^ ^2^ Since language delay can be an indicator for several neurodevelopmental problems, it should be taken seriously and further diagnostic investigation is recommended.^3^

There is general agreement regarding the need for intervention for children with persistent deficits in expressive language in the late preschool period and children with deficits in receptive language.^4^ ^5^ But in anticipation that young children with specific expressive language delay (SELD) have a good prognosis and will normalise spontaneously, the “wait and see” strategy is widely recommended,^4^^–^6 and speech and language therapy is usually not initiated before the age of 4 years.^7^ The main reason for this approach is that language development is still quite variable in typically developing young children, and it has been shown that many children with SELD normalise, that is, their language skills at the age of 3–5 years are similar to those of their peers.^6^

**What is already known on this topic**

- Although a substantial group of children with expressive language delay will not resolve their problem spontaneously, the “wait and see” approach is widely used.
- Parent based language intervention and child directed intervention are effective, but the established programmes are costly and very time consuming.
- Evaluated parent based intervention programmes are not available in German speaking countries.

**What this study adds**

- This randomised controlled trial shows that a highly structured and very short parent based language intervention group programme is effective.
- The Heidelberg Parent-based Language Intervention is less expensive and time consuming than other published parent based language interventions.

However, the picture is not entirely clear as other studies have found that at least 50% of children with SELD do not resolve their problem spontaneously.^8^^–^12 Studies that started with preschool children with follow-up into school age and adolescence, have found that a substantial proportion of children, in particular those with receptive language impairments, will not outgrow their language difficulties and are therefore at risk for cognitive, literacy, behavioural and psychiatric problems.^13^^–^15

This unfavourable long term prognosis, combined with parental concerns, child frustration and disturbed parent–child interactions,^16^ has led to the development of early intervention models such as individual directed interventions, combined parent–child language groups or parent based group interventions. A limited number of intervention studies have been published.^17^^–^22

Different parent based group approaches are effective^19^^–^22 and well established in North America and the UK, but they do not seem to be cheaper or less time consuming than individual directed interventions.^21^ Evaluated parent based intervention programmes are not available in German speaking countries.

The purpose of this study was to examine the effectiveness of a short and highly structured parent based language intervention group programme for 2-year-old children with SELD. The main hypothesis was that 2-year-old children with SELD whose mothers participate in the intervention, will show improved expressive language abilities 6 and 12 months after pretest in comparison to children with SELD in a waiting group. The results of both clinical groups were compared to the results of a matched reference language-normal sample. Confirmation of significant intervention effects might have substantial practical implications for providing support for children with SELD.

## METHOD

### Study design

The randomised controlled trial (RCT), completed in the Children’s Hospital, University of Heidelberg, utilised a pretest–post-test control group design with follow-up 12 months after pretest.

Based on three frequently cited studies, a power calculation for single sided t tests was used to determine the sample size.^19^ ^23^ ^24^ If α was set at 0.05 and β was 0.80, 14 subjects per group were required. It was decided to aim for a sample size of 20 subjects per group.

Randomisation was carried out sequentially after pretest to achieve a balanced parallel group design stratified for gender and maternal school education because earlier studies found a correlation between maternal education and language development at the age of 3 and 4 years.^11^ Randomisation was carried out using opaque sealed envelopes. Post-test and follow-up diagnostics were carried out by different assessors who were blinded to previous results and allocation.

The study received ethical approval from the Ethics Committee of the University of Heidelberg.

### Participants

Sixty one children with SELD were selected from a sample of children with language delay, identified in general paediatric practices during free routine developmental check-ups at 21–24 months of age from October 2003 to February 2006.^3^ Inclusion criteria were singletons born at term without pre-, peri- or postnatal complications and a German speaking family background. Exclusion criteria were chronic hearing deficits, persistent middle ear effusion accompanied by a significant hearing loss of >20 dB, visual impairments, genetic syndromes, pervasive developmental disorders or other diseases with a known influence on language development, deficits in receptive language and/or in non-verbal cognitive abilities, and previous language intervention. The children were between 24 and 27 months of age at entry into the study (mean age 24.7 months, SD 0.9). None of these children had reached the critical cut-off of 50 words in their expressive vocabulary as measured by the parent-report screening questionnaire ELFRA-2.^25^

**Figure 1:**
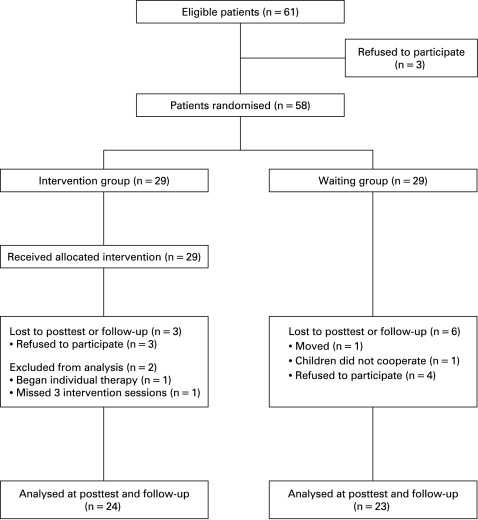
Flowchart of patient involvement in the study.

Nine families dropped out before follow-up. Two families from the intervention group were excluded from analysis, resulting in a final sample of 47 children with SELD (fig 1).

To obtain a reference language-normal group, an advertisement was placed in a local newspaper. A total of 36 children (mean age 24.6 months, SD 0.8) were matched as closely as possible with respect to age, sex, birth order and maternal school education.

Demographic and clinical data are presented in table 1.

**Table 1:** Demographic and clinical data of children in the intervention group, the waiting group and a reference language-normal group

	Intervention group, n = 24(13 males/11 females)	Waiting group,n = 23(11 males/12 females)	Fisher’s exact test*	LN group,n = 36(20 males/16 females)
Birth order, %			0.22 NS	
First born	20.8	43.5		41.7
Second born	62.5	39.1		50.0
Third or fourth born	16.7	17.4		8.3
Family history of SLD (1st degree), %	50.0	43.5	0.77 NS	2.8
Age of mothers at birth, mean (SD), years:months	32:1 (3:8)	33:7 (4:3)		31:7 (4:4)
Maternal school education (years in school), %			0.58 NS	
No/low graduation (8–9)	12.5	8.7		11.1
Middle school graduation (10)	37.5	56.5		47.2
High school graduation (13)	50.0	34.8		41.7
Maternal work situation, %			0.57 NS	
Full time employment	8	4		3
Part time employment	38	52		44
House wife	54	44		53

*Comparison of intervention and waiting group.

LN, language-normal; NS, not significant; SLD, speech and language disorder.

### Measures

During the routine paediatric check-up, parents completed the ELFRA-2 (the German version of the MacArthur Communicative Development Inventories),^26^ a reliable and easy to use parent-report screening questionnaire for the early identification of children with language delay.^27^

At pretest, children were tested with the widely used developmental language test for 2-year-old children (SETK-2), a standardised and norm-referenced instrument to examine the language status of German speaking children.^28^ Two subtests measure language comprehension and two subtests measure word production (naming objects/pictures) and sentence production (explaining pictures). Cognitive abilities were assessed with the Mental Scale of the Bayley Scales of Infant Development, 2nd edition, Netherlands version (BSID-II-NL) which offers norms for a general mental developmental index (MDI) and for a non-verbal MDI.^29^ Only children with normal non-verbal cognitive abilities (non-verbal MDI>85) were included. Audiometric testing was carried out for all children to exclude those with persistent middle ear effusion accompanied by a significant hearing loss of >20 dB. A neurological examination was performed by a paediatric neurologist to exclude children with neurological diseases.

At post-test, expressive language was standardised assessed with the two production subtests of the SETK-2; in addition, parents completed the ELFRA-2 questionnaire.

At follow-up, expressive language was tested with the two production subtests of the SETK 3–5, a standardised and norm-referenced instrument to examine the language status of German speaking preschool children (reliability coefficients 0.62–0.86).^30^ Language production was measured with the subtest Encoding Semantic Information (ESI) in the same manner as with the two production subtests of the SETK-2. The other subtest measures the ability of plural forming (PF). Parents completed the ELFRA-2 questionnaire research version, which includes six additional syntactic items for better differentiation of 3-year-old children.^25^

Results within normal limits (t score ⩾40) in both production subtests of the SETK 3–5 indicated the child had caught up. Specific language impairment was defined by a t score of more than 1.5 SD below the mean (⩽35) in at least one production subtest of the SETK 3–5.

All diagnostic sessions were recorded on videotape.

### Intervention programme

The intervention used was the Heidelberg Parent-based Language Intervention (HPLI),^31^ ^32^ a highly structured and interactive programme developed for use with a group of 5–10 parents. The 3-month programme consisted of seven 2 h and one 3 h session 6 months later.

The HPLI is based on an interactive model of language intervention, which presumes that optimised parental input will provide better language learning opportunities for children.^33^ Parents are introduced to child oriented, interaction promoting and language modelling techniques.^34^ Sharing picture books is one of the main topics of the programme, since picture book sharing is an ideal time to initiate communication as well as being a prototypical situation for learning words at the age of 2.^24^

The intervention started when the children were about 25 months old. To achieve comparability only mothers took part; about seven mothers took part in each group. All sessions took place at the Children’s Hospital, University of Heidelberg and were conducted by the first author who had developed the HPLI.

### Statistical analysis

Statistical analysis was performed using SAS (v 8.01). Frequency differences between groups were tested for using the χ^2^ or Fisher’s exact test. Pretest comparisons were made using analysis of variance (ANOVA) followed by two sided t tests. A series of ANOVAs, single sided t tests, calculation of effect sizes (Cohen’s d)^35^ and repeated measurements MANOVAs (using the non-verbal MDI as a covariate) were administered to test for treatment effects. All hypotheses were directional, so the one tailed probability level was set at 0.05.

## RESULTS

The results are presented in three sections below: (1) pretest, (2) post-test and (3) follow-up comparisons of the intervention group, waiting group and reference language-normal group.

### Pretest comparisons

The three groups differ significantly on all language scores and on general mental abilities (ANOVA, table 2). The intervention and waiting groups did not differ significantly on any of the demographic data (table 1) or on any language score (table 2). Language comprehension was age appropriate in both clinical groups. The language-normal group differ significantly from the intervention and waiting groups on all language scores (table 2) and on the variable family history of speech and language disorder (SLD) (χ^2^ test, p<0.001).

**Table 2:** Pretest comparisons on language and cognitive abilities

Pretest	Inter-vention group, n = 24	Waiting group, n = 23	LN group, n = 36	ANOVA		Comparison of intervention and waiting groups		Comparison of intervention and LN groups		Comparison of waiting and LN groups	
	Mean (SD)	Mean (SD)	Mean (SD)	F value¶	p Value	t Score**	p Value	t Score	p Value	t Score	p Value
Assessment of language abilities											
ELFRA-2 (parent report)*											
Age in months	23.9 (1.0)	24.5 (0.8)	24.2 (0.7)	F(2,80) = 2.9	0.062	−2.2	0.034	−1.5	0.155	1.1	0.263
Vocabulary	16.6 (8.9)	14.3 (9.9)	160.9 (44.2)	F(2,80) = 238.9	<0.001	0.8	0.404	−19.0	<0.001	−19.2	<0.001
Syntax	1.0 (2.0)	1.0 (2.1)	24.9 (9.1)	F(2,80) = 150.3	<0.001	−0.1	0.942	−15.2	<0.001	−15.1	<0.001
Morphology	0 (0)	0 (0)	8.0 (3.8)	F(2,80) = 104.5	<0.001	−	–	−12.7	<0.001	−12.7	<0.001
SETK-2†											
Age in months	24.5 (0.9)	24.9 (0.9)	24.6 (0.8)	F(2,80) = 1.3	0.277	−1.5	0.130	−0.9	0.395	0.9	0.369
Comprehension‡											
Words	52.2 (8.8)	50.9 (5.6)	56.3 (7.2)	F(2,80) = 4.6	0.015	0.6	0.561	−1.9	0.064	−3.2	0.002
Sentences	51.0 (7.9)	49.0 (7.5)	58.4 (10.4)	F(2,80) = 9.2	<0.001	0.9	0.390	−3.1	0.003	−4.0	<0.001
Production‡											
Words	31.1 (2.7)	30.7 (3.8)	57.4 (8.8)	F(2,80) = 177.0	<0.001	0.5	0.657	−16.6	<0.001	−16.0	<0.001
Sentences	37.2 (2.9)	35.9 (4.1)	55.0 (7.4)	F(2,80) = 113.9	<0.001	1.3	0.218	−13.1	<0.001	−12.8	<0.001
Assessment of cognitive abilities											
BSID-II-NL§											
MDI	96.2 (6.9)	95.3 (8.1)	114.3 (10.5)	F(2,80) = 43.7	<0.001	0.4	0.714	−8.0	<0.001	−7.8	<0.001
Non-verbal MDI	115.2 (10.1)	109.6 (12.6)	116.5 (10.5)	F(2,80) = 2.9	0.063	1.7	0.101	−0.5	0.64	−2.2	0.034

^*^Raw score; †SETK-2, for description of this language test see Buschmann *et al*^3^; ‡t score normative means are 50 (SD 10); §standard score normative means are 100 (SD 15); ¶ANOVA over all three groups; **two sided t test adjusted for multiple testing using Bonferroni corrections.

BSID-II-NL, Bayley Scales of Infant Development, 2nd edn, Netherlands version; ELFRA-2, [Parent report screening questionnaire for early identification of children at risk]; LN, language-normal; MDI, mental developmental index; SETK-2, [Developmental language test for 2-year-old children].

### Post-test comparisons

At post-test all three groups showed an improvement in parent reported language scores. The language-normal group scored significantly higher compared with the clinical groups on all language scores (table 3). Children in the intervention group demonstrated greater gains than children in the waiting group in parent reported vocabulary, morphology, syntax as well as in both production subtests of the SETK-2, with medium to very large effect sizes (table 3). Regarding mean t scores, both clinical groups showed improvement in the subtest word production. However, in the subtest sentence production, only the intervention group improved their mean t score (tables 2 and 3).

**Table 3:** Post-test and follow-up comparisons on language abilities

	Intervention group, n = 24		Waiting group, n = 23		LN group, n = 36		ANOVA		Comparison of intervention and waiting group			Comparison of intervention and LN group		Comparison of waiting and LN group	
	Mean (SD)	95% CI‡	Mean (SD)	95% CI	Mean (SD)	95% CI	F value§	p Value	t Score	p Value¶	d**	t Score	p Value	t Score	p Value
Post-test															
Age in months	30.5 (0.9)		31.0 (1.0)		30.6 (0.6)		F_(2,80)_ = 1.9	0.156	−1.5	0.134	–	−0.2	0.844	1.6	0.115
ELFRA-2 (parent report)*															
Vocabulary	140.7 (57.3)	117 to 165	96.3 (64.0)	69 to 124	219.5 (26.8)	210 to 229	F_(2,80)_ = 48.1	<0.001	2.5	0.016	0.73	−6.3	<0.001	−8.8	<0.001
Syntax	22.9 (7.1)	20 to 26	13.5 (9.0)	10 to 17	40.2 (8.9)	37 to 43	F_(2,80)_ = 76.3	<0.001	4.0	<0.001	1.16	−8.4	<0.001	−11.2	<0.001
Morphology	7.0 (4.2)	5 to 9	4.0 (4.1)	2 to 6	13.9 (2.8)	13 to 15	F_(2,80)_ = 58.5	<0.001	2.5	0.017	0.72	−7.0	<0.001	−10.2	<0.001
SETK-2†															
Word production	49.4 (10.2)	45 to 54	41.0 (12.4)	36 to 46	56.9 (8.4)	54 to 60	F_(2,80)_ = 17.3	<0.001	2.5	0.016	0.74	−3.0	0.005	−5.4	<0.001
Sentence production	41.0 (5.3)	39 to 43	35.3 (5.8)	33 to 38	56.8 (11.1)	53 to 61	F_(2,80)_ = 52.2	<0.001	3.5	0.001	1.03	−7.4	<0.001	−9.7	<0.001
Follow-up															
Age in months	37.3 (1.3)	36 to 40	38.0 (1.7)	36 to 42	37.4 (1.2)	36 to 40	F_(2,80)_ = 2.1	0.125	−1.6	0.114	–	0	0.967	1.7	0.090
ELFRA-2 (parent report)*															
Vocabulary	216.9 (36.9)	91 to 260	178.0 (65.6)	18 to 260	246.0 (16.5)	240 to 252	F_(2,80)_ = 19.2	<0.001	2.5	0.018	0.73	−3.6	0.001	−4.9	<0.001
Syntax	43.3 (12.0)	21 to 59	34.4 (18.9)	0 to 65	56.6 (4.0)	55 to 58	F_(2,80)_ = 24.7	<0.001	1.9	0.062	0.67	−5.2	<0.001	−5.5	<0.001
Morphology	12.5 (3.3)	4 to 16	9.2 (5.7)	0 to 16	15.5 (1.0)	15 to 16	F_(2,80)_ = 22.6	<0.001	2.4	0.021	0.71	−4.4	<0.001	−5.3	<0.001
SETK 3–5†															
Encoding Semantic	51.6 (11.2)	32 to 81	43.9 (9.1)	27 to 62	60.2 (10.4)	57 to 64	F_(2,80)_ = 17.8	<0.001	2.6	0.013	0.75	−3.0	0.005	−6.3	<0.001
Information, ESI															
Plural Forming, PF	48.0 (9.3)	30 to 67	45.5 (10.0)	30 to 62	57.1 (8.9)	54 to 60	F_(2,80)_ = 13.0	<0.001	0.9	0.376	0.23	−3.8	<0.001	−4.5	<0.001

*Raw score; †t score normative means are 50 (SD 10); ‡95% confidence interval; §ANOVA over all three groups; ¶single sided t tests; **effect size, Cohen’s d.^31^

ELFRA-2, parent report screening questionnaire for early identification of children at risk; LN, language normal; SETK 3–5, [Developmental language test for 3- to 5-year-old children].

### Follow-up comparisons

#### Between groups

Twelve months after pretesting all three groups showed a further increase in parent reported language scores. The language-normal group scored significantly higher compared with the clinical groups on all language scores. Significant group differences between the intervention and waiting groups were found for parent reported vocabulary and morphology as well as for the subtest Encoding Semantic Information (ESI) (table 3).

**Figure 2:**
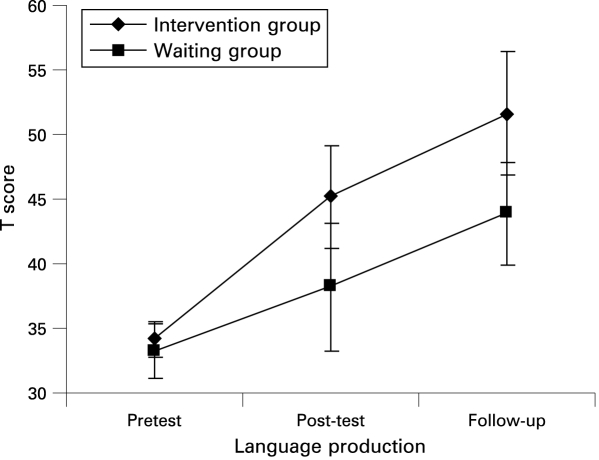
T scores (mean value, 95% confidence interval) for language production at pretest, post-test and follow-up for the intervention and waiting groups.

Figure 2 shows the means and the 95% confidence intervals for language production test scores at pretest, post-test and follow-up for the intervention and waiting groups. The two production subtests of the SETK-2 were combined at pretest and post-test. At follow-up the subtest Encoding Semantic Information (ESI) was used for analysis. A repeated measurements MANOVA (with the non-verbal MDI as covariate) showed a significant main effect for group (F(1, 97) = 8.23, p = 0.006) as well as a significant interaction between group and tests (F(2, 39) = 3.80, p = 0.026), but not a significant test effect (F(2, 39) = 2.26, p = 0.11) for language production at follow-up. For the subtest Plural Forming (PF), a group effect (F(1, 92) = 2.84, p = 0.1) and an interaction between group and tests (F(2, 33) = 2.65, p = 0.08) were found, but they failed the 0.05 significance level. There was no test effect (F(2, 33) = 0.31, p = 0.74) (adjusted by Greenhouse-Geisser).

#### Across time

For language production a very significant time effect between pretest, post-test and follow-up (F(1, 29) = 51.16, p<0.001) and an interaction between time and group (F(1, 29) = 7.59, p = 0.008) was revealed.

### Number of individuals who “caught up”

At follow-up, 18 children (75%) in the intervention group, 10 children (43.5%) in the waiting group and all children in the language-normal group showed results within the normal limits (t score ⩾40) for expressive language. In the intervention group, two children (8.3%) fulfilled the diagnosis of specific language impairment (t score ⩽35) versus six children (26.1%) in the waiting group and none in the language-normal group. There was no significant difference regarding gender (1) or maternal school education (2) between intervention group children who “caught up” in comparison to those who showed continuing impaired expressive language ((1) p = 0.17, (2) p = 0.29, Fisher’s exact test) or between children in the waiting group who caught up compared to children with persistent impaired expressive language ((1) p = 0.21; (2) p = 0.67).

### Cost effectiveness

The HPLI costs £270 per child. In Germany the cost of one individual directed therapy session is £28. Because individual therapy for children with specific language impairment takes an average of 43 sessions,^7^ the labour costs amount to £1204 per child. As six children in the intervention group needed individual directed therapy, the labour costs amounted to £13 704 for the whole intervention group, including the cost of the HPLI and the expected cost for additional individual directed therapy for the six children. In the waiting group 13 children needed individual therapy, so the expected cost was £15 652.

## DISCUSSION

In this RCT we examined the effectiveness of the HPLI in a group of 2-year-old children with SELD. The results support previous evidence that early parent based language intervention is effective in the short term.^19^ During the 6- and 12-month intervals, children in the intervention group made developmental gains in vocabulary and grammatical abilities over and above the maturational changes seen in the waiting group. However, the most important result was that the percentage of children who showed standardised scores within normal limits in expressive language and therefore had caught up with their peers at the age of 3, was 75% in the intervention group in contrast to 44% in the waiting group. Thus, the percentage of children who needed to start additional individual directed language therapy was significantly lower in the intervention group compared to the waiting group. These differences are suggested to be the result of participating in a highly structured and short parent based language intervention. These findings support previous evidence that the interactive style of mothers may be optimised to provide a superior language learning environment and accelerate the language development of late-talking toddlers.^19^

Since the subjects showed only expressive language delay, the results cannot be generalised to children with additional deficits in receptive language or to children with concurrent cognitive deficits. In addition, despite impressive improvement due to our intervention, the expressive language abilities of the intervention group remained significantly lower compared to the language-normal group.

The importance of the HPLI as an effective prevention programme is underscored by the fact that there is only little evidence for the effectiveness of individual therapy implemented in preschool children.^36^ Compared to the established Hanen Parent Programme (HPP),^37^ the HPLI offers a more structured approach, takes a shorter time and is less expensive and time consuming.^21^ It is carried out without home visits by a single HPLI-trained therapist.

The results of our study have important clinical implications for providing support for children with SELD. Currently, parent report screening questionnaires such as the MacArthur Communicative Development Inventories are seldom used in German speaking countries to identify children with language delay, even though they can be easily used in general paediatric practice.^27^ One reason for their low acceptance could be that currently there are no guidelines on how to most appropriately provide support for children with language delay. While the wait and see approach is widely used, it cannot be recommended on the basis of our results for the following reasons. First, the heterogeneity of children with language delay makes further diagnostic work-up necessary.^3^ Second, the persistence of language impairments in a substantial number of children together with related educational, social-emotional and behavioural problems clearly indicates the need for early language intervention that helps children develop normal linguistic functioning as quickly as possible. One possible approach is a parent based intervention with the advantage that parents are perceived to be competent partners in the facilitation of language development. According to our results, the HPLI also seems to be successful in families with low socio-economic status.

The results of this RCT show that the HPLI is an effective and cost saving approach in providing support for children with SELD. Further follow-up investigations are necessary to evaluate the long term effectiveness of the HPLI.
